# Transmogrification of ocean into continent: implications for continental evolution

**DOI:** 10.1073/pnas.2122694119

**Published:** 2022-04-04

**Authors:** Jason P. Morgan, Paola Vannucchi

**Affiliations:** ^a^Department of Marine Science and Engineering, Southern University of Science and Technology, Shenzhen, Guangdong 518055, China;; ^b^Department of Earth Sciences, University of Florence, 50121 Florence, Italy

**Keywords:** continental crust and lithosphere, ocean lithosphere, origin of continents

## Abstract

When continents collide, the typical embayments and protrusions along their rifted margins make it likely that fragments of seafloor will be trapped within the growing orogenic belt. These trapped seafloor fragments become preferential depocenters for marine and terrestrial sedimentation. After ∼0.5 Gy, the high radioactivity of their thick terrigenous sediment pile converts former seafloor into a unique form of continental crust and underlying lithosphere. We call this process transmogrification. Initially strong and low-lying basins that act as mechanically stronger blocks in the collisional orogeny will eventually warm, weaken, and thermoisostatically rise and will eventually transform into preferred sites for future continental rifting. In modern Asia, transmogrifying basins have induced the characteristic paired-mountain belt geomorphology associated with the assembly of this supercontinent, for example, the Himalaya/Tibet + Tian Shan surrounding the Tarim Basin that has greatly strengthened the East Asian Monsoon. The time-dependent temperature, uplift, and strength changes associated with transmogrification are relevant for improving our understanding of continental evolution, basin modeling, paleoclimate studies, and natural resources prospection.

## Significance

This study proposes a geological mechanism for creating continental crust and lithosphere. When continents collide, the typical embayments and protrusions along their rifted margins make it likely that fragments of seafloor will be trapped within the growing mountain belt. These become preferential centers of sedimentation that eventually convert former seafloor into a unique form of continental crust and lithosphere, leading to characteristic temporal changes in the relative strength and uplift/subsidence of these regions. The Alpine–Himalayan mountain chain in Eurasia shows several stages of this process, which we further explore with a one-dimensional thermal-rheological model. In Asia, this process appears to have created a characteristic paired-mountain belt geomorphology (Himalaya/Tibet + Tian Shan) that has greatly strengthened the East Asian Monsoon.

The growth and evolution of continental crust has played an important role in Earth’s chemical differentiation through time. Less attention has been given to the processes that create and destroy continental lithosphere. To balance the inevitable destruction of subcontinental mantle lithosphere during the continent–continent collisional phase in the Wilson Cycle, continental lithosphere must also have been created, as both the volume and surface area (freeboard) of continental crust have remained relatively constant over Earth’s history, implying that continental crustal thickness and crustal area and the surface area underlain by subcontinental lithosphere have all stayed relatively constant over the past ∼2.5 to 3 Ga (1–5). Motivated by the current geology and tectonics of Eurasia, we propose a mechanism in which continent–continent collisions are directly linked to the creation of new continental lithosphere. We start from the hypothesis that when two continents collide, not all intervening oceanic lithosphere subducts, at least not every time. When oceanic lithosphere becomes buried by tens of kilometers of marine and terrestrial sediments, it will be converted—transmogrified—into continental crust and lithosphere. Here, we study this process.

**Fig. 1.**
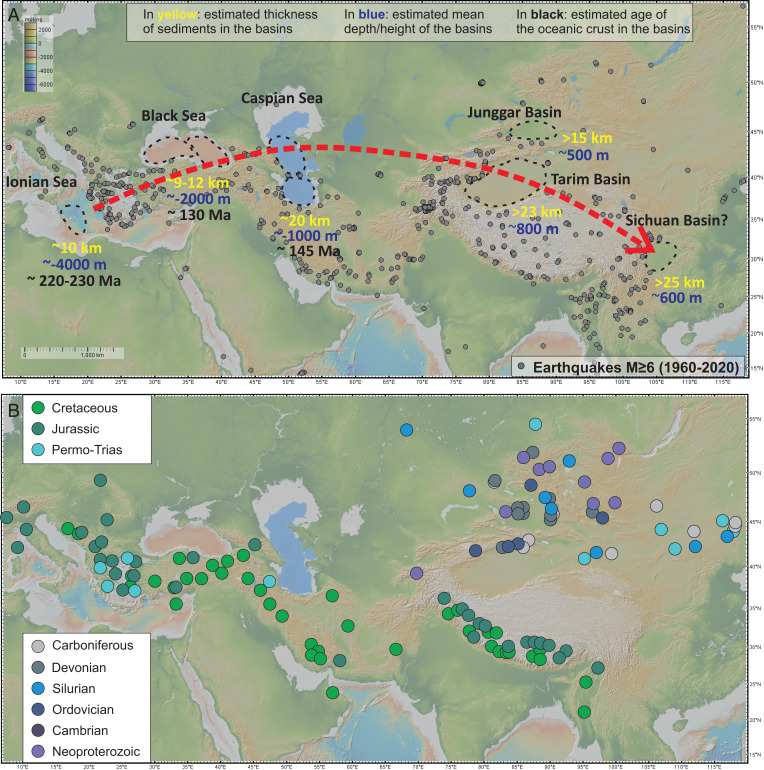
Map of Eurasia showing basins proposed to currently be in different stages of the transmogrification process. (*A*) Dashed contours outline the Ionian Sea, Black Sea, Caspian Sea, Tarim Basin, Junggar Basin, and Sichuan Basin. Magnitude > 6 earthquakes (gray circles) avoid these basins. Yellow numbers show the estimated thicknesses of sediments in these basins, blue numbers show their current average relief, and black numbers show the seafloor age (where available) beneath the basin. (*B*) Ages and location of ophiolites in the Alpine–Himalayan (Cretaceous, Jurassic, and Triassic) orogeny and the older, but still ongoing, Central Asian Orogeny delineate the Eurasian regions involved in continent–continent and oceanic terrane–continent collisions since the Neoproterozoic. Ophiolite age–location data after refs. 51 and 52.

Usually continent–continent collision is visualized as a two-dimensional (2D) cartoon cross-section in which all intervening oceanic lithosphere subducts. This idealization neglects the fact that most rifted continental margins are associated with significant geometric embayments and protrusions with respect to an idealized 2D cross-section. This geometry is clearly seen in reconstructions of the Tethyan collision associated with the modern Alpine–Himalayan orogeny. Natural geometric irregularities mean that collision does not happen at the same time over the whole continental margin but starts in some areas while adjacent areas still subduct oceanic lithosphere. A key question is whether the “left-over” portions of the margins’ oceanic lithosphere will ever completely subduct. In Eurasia, for example, Black Sea and Caspian Sea (Fig. 1) portions of Neotethys on the overriding plate are now surrounded by “continental” crust.

## Results

### The Eurasian Record of Tethyan Transmogrification.

Eurasia preserves geologic snapshots of several stages in the transmogrification process of Tethyan seafloor. The earliest stage is seen in the Mediterranean’s Ionian Sea, a deep basin that seems destined to survive the final stages of African/Eurasian collision, as subduction has essentially stopped with modern convergence rates being <3 to 5 mm/y (6). The seafloor is currently ∼4,000 m deep, with ∼7 km of ∼230 to 250 My-old Neotethyan ocean crust overlain by ∼6 km of mostly continentally derived sediments (7). The second stage is seen in the Black Sea. Here, the seafloor reaches ∼2,000-m depths, with ∼130 My-old Paleotethyan ocean crust and lithosphere now overlain by ∼9 to 12 km of continental sediments (8, 9) surrounded by orogenic belts containing both arc and continental crust. The third stage, again involving Paleotethyan oceanic crust, is seen in the South Caspian Sea. This relic seafloor is currently ∼1,000 m deep and now consists of ∼7 km of oceanic crust overlain by ∼14 to 18 km of continentally derived sediments (10). It also laterally abuts more deformed arc and continental crust at its margins. While researchers have previously associated the above examples with former ocean seafloor, the identification of later stages of transmogrification is more speculative, as it involves thickly sedimented basins. Possible examples are the Tarim Basin and Junggar Basin in Central Asia and maybe even the Sichuan Basin in East Asia. These basins have been previously called Chinese type (11) or plate interior poly-phase basins (12). In these, enough sediment fill has occurred to create a ∼35- to 40-km–thick crustal package (13). In the Junggar Basin, two recent seismic interpretations favor a basement made of an oceanic plateau with thickened oceanic crust (14, 15). We speculate that in the Tarim Basin, ∼7 to 10 km of oceanic crust may be currently overlain by ∼25+ km of sediments. There is a wide range of postulated Tarim basement materials that range from a late Precambrian–early Paleozoic failed rift (16) to arc basement (17), to oceanic plateau (18), to ocean crust (19). However, the only drill site that reached basement penetrated an arc-like diorite located on a basement high (17). This location and its morphology are similar to the basement ridges of the Mid Black Sea high (9). In the Black Sea, these form the western boundary of an even older oceanic crust–floored basin. This similarity suggests that the Tarim might also have older oceanic basement in its deepest regions.

### Thermal Model of Transmogrification.

We use a one-dimensional (1D) thermomechanical model to explore the thermal and mechanical evolution that takes place during the transmogrification of ocean into continent. A finite difference–based thermal model is used to determine the thermal evolution of ocean seafloor that is progressively buried by first a marine then later a subaerial sediment load. The resulting compositional and temperature profiles are used to determine the evolution of strength, heat flow, and isostatic uplift using standard density (20) and rock strength (21) parameterizations. The brittle ductile yield strength envelope is determined using the approach and 10^−15^ s^−1^ assumed strain rate used by Kohlstedt et al. (21) to make a first-order lithospheric strength characterization. We used the brittle and viscous parameterizations of Ros et al. (20) for sediment/upper crust (“quartz-like”), oceanic/lower crust (“mafic granulite-like”), and mantle (“olivine-like”) rheologies (*Materials and Methods*).

**Fig. 2.**
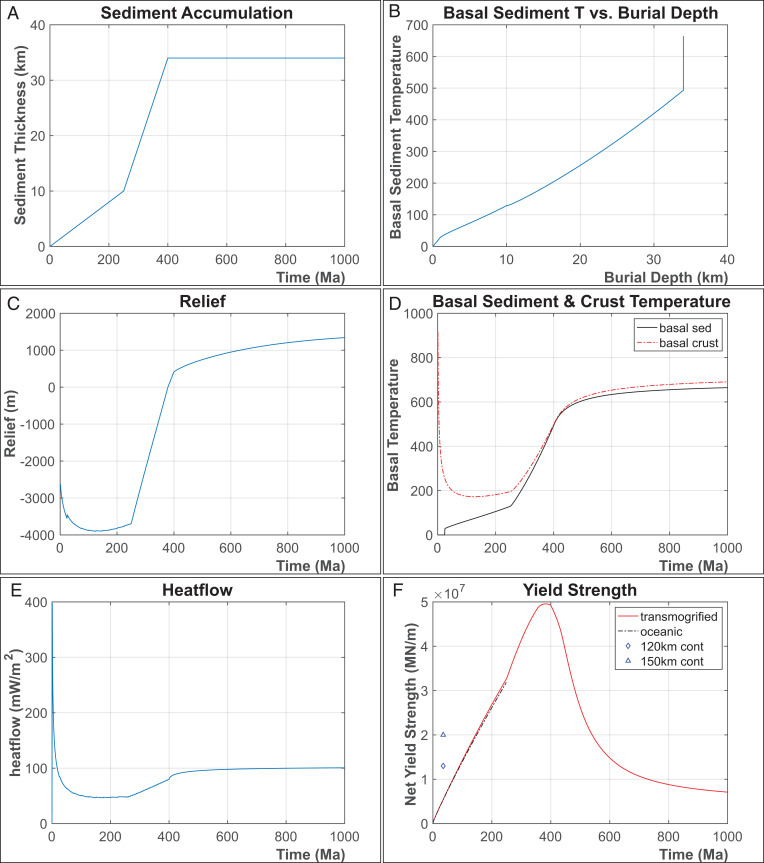
Panels illustrating thermal, sedimentary, and mechanical changes that take place during an exemplar of the transmogrification process. (*A*) Sediment accumulation over 1 Ga of transmogrification. (*B*) Basal sediment temperature versus burial depth. (*C*) Evolving surface relief. (*D*) Basal sediment (black) and basal crust (red) temperature versus time. (*E*) Heat flow over time. (*F*) Integrated yield strength versus time. Diamond and triangle symbols show comparative integrated yield strengths for continents with steady-state 120-km and 150-km lithosphere thicknesses.

**Fig. 3.**
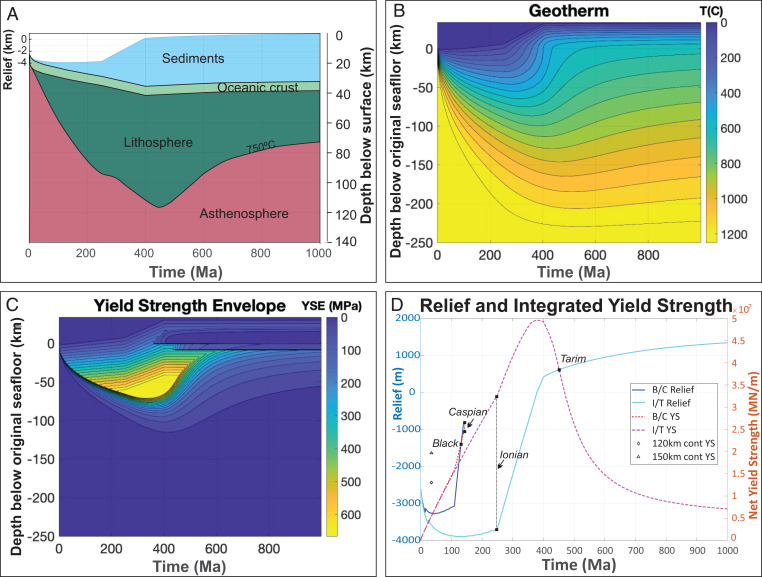
(*A*) Depth–time cross-section summarizing the geometric, sedimentary, and thermal evolution during the transmogrification exemplar shown in Fig. 2. Here, the lowermost (oldest) sediments deposited are deep marine sediments, while later sedimentation becomes continental in provenance and then even subaerial after the basin has become an interior continental basin surrounded by high topography. The time-evolving depth transition between lithospheric mantle and asthenosphere is temperature controlled, as shown in *B* and *C*. (*B*) Depth (with respect to orig. seafloor), temperature versus time for the exemplar shown in Fig. 2. (*C*) Depth (with respect to orig. seafloor), yield strength versus time for the exemplar shown in Fig. 2. (*D*) Integrated yield strength and relief versus time for scenarios illustrating the transmogrification paths of the Ionian, Black, and Caspian Seas and the Tarim Basin. Black squares show the present-day predicted integrated yield strength and relief of these basins. The *Materials and Methods* section contains more information on the parameters used in these numerical experiments.

The exemplar illustrated in Figs. 2 and 3 shows the thermomechanical consequences of the thermal evolution of ocean crust that first ages for 250 My—as the Ionian seafloor has done—while being loaded by continentally derived sediments at a rate of 10 km/250 My. After this time, the oceanic crust–floored basin is assumed to have become trapped within an Alpine–Himalayan-like orogenic belt, where it is surrounded by growing boundary relief (discussed later), so that its sediment accumulation rate would increase to 24 km/150 My over the next 150 My (Fig. 2*A*). If this loading phase is rapid enough—as it is in the exemplar in Fig. 2 from 250 to 400 My—then the loading effect of sediments on increasing the lithosphere’s Mohr–Coulomb yield strength causes the lithosphere to initially strengthen during a “rapid” phase of basin infill (Figs. 2*F* and 3*C*). After 24 km of additional basin infill, 34 km of sediment accumulation has led to the still cold but warming basin becoming subaerial, analogous to a subaerial Tarim-like internal basin stage of transmogrification. Eventually, after ∼140 My of rapid infilling in the exemplar in Fig. 2, the high radiogenic heat productivity of the basin’s continent-derived sediment load causes the basin’s underlying crust and mantle to slowly continue to warm (Fig. 2 *B* and *D*) and weaken (Fig. 2*F*), which leads to slow thermoisostatic epeirogenic basin uplift (Fig. 2*C*) and an eventual strength inversion with respect to neighboring noncratonic continental lithosphere (Fig. 2*F*). Figs. 2 and 3 summarize the crustal and lithospheric changes during this evolution. During the later stages of its thermal evolution, radiogenic lower crust will metamorphose and partially melt (Fig. 2 *B* and *D*), at which point upwardly migrating melts/fluids would preferentially transport its radiogenic elements toward the surface where they will have less of a warming effect on underlying crust and mantle. Once weaker than surrounding continental material, transmogrified crust and its underlying lithosphere would preferentially become incorporated into orogenic belts, where it would be destroyed through uplift and erosion of its crust and deformation/extrusion/subduction of its now-weak mantle lithosphere in preference to other types of continental lithosphere. However, a continent may even experience two Wilson cycles in the ∼500-Ma time interval during which transmogrified basins slowly evolve to become relatively weak sites of preferred rifting and orogenesis.

## Discussion

### On the Relative Importance of Transmogrification in Building Continental Lithosphere.

The implied minimum net rate of Tethyan transmogrification can be estimated from the net surface area (∼1 × 10^6^ km^2^) of the presumed “ocean-floored” Ionian Sea, Black Sea, Caspian Sea, Tarim, and Sichuan Basins (black-circled regions in Fig. 1) divided by the inferred ∼184-My closure time of the Paleotethys and Neotethys provided by the ∼184-My age of the earliest Karoo–Ferrar flood basalt stage of Gondwana rifting (22) (∼185 My is also the age of the early Toarcian anoxic event and Pliensbachian–Toarcian extinction). This inference leads to an estimated rate of ∼5 × 10^3^ km^2^/My or 3.1% continental area (CA)/Gy (CA = 148 × 10^6^ km^2^). This transmogrified area is comparable to the current area of the high Tibetan Plateau (∼1.6 × 10^6^ km^2^), although the recent ∼50- to 0-My net destruction rate of Indian mantle lithosphere beneath Tibet is higher (∼3 × 10^4^ km^2^/My) than the inferred average long-term rate of transmogrification. If we consider the potential transmogrification of potential future trapped ocean crust–floored basins, such as the Bengal Fan (9 × 10^5^ km^2^) and Gulf of Mexico (2.7 × 10^6^ km^2^), then the above estimate for recent global transmogrification rates would be increased by ∼two- to fourfold.

This estimate for the modern rate of transmogrification is a geologically significant fraction of the inferred rates of other proposed processes that could create continental lithosphere. One such process is the gravitational spreading force associated with overthickened crust that would induce it to spread once subduction stops, as may have happened during the formation of the Basin and Range at the end of the Laramide orogeny (23, 24). This rate can be estimated by dividing the “newly” extended area (4 × 10^5^ km^2^) of the Basin and Range (25) by the 60 My since the end of the Laramide Orogeny, which yields 6.7 × 10^3^ km^2^/My (4.5% CA/Gy). More recently, McQuarrie and Wernicke (26) estimated a slightly lower minimum extended area of 235 ± 20 km × 1,500 km = 3.5 × 10^5^ km^2^. The potential creation of new continental crust and lithosphere at volcanic arcs appears to be up to an order of magnitude larger. This rate is particularly difficult to estimate because subduction zones are places where forearc crust and mantle are both added (27) and removed by subduction erosion (28). Because this rate is so hard to assess, Clift et al. (29) chose to first estimate all other rates of continental crustal addition and removal and then assume that the recent rate of crustal production associated with arc magmas is the difference between all other better-known rates of crustal loss and production, for example, the rate needed to maintain the volume of the continental crust since the Permian. Table 8 of Clift et al. (29) summarized that net removal rates of continental crust have been ∼4.93 km^3^/y since the Permian, while other sources of crustal production have been the accretion of oceanic plateaus (∼1.1 km^3^/y) and the addition of continental large igneous provinces (∼0.03 km^3^/y). This led to an inferred rate for the addition of arc magmas of ∼3.8 km^3^/y (= >4.93 km^3^/y – 1.13 km^3^/y). If we assume that arc crust is 38 km thick (29), then a 3.8-km^3^/y rate for the addition of arc magmas would imply an average rate of continental lithospheric production of 0.1 km^2^/y (or 10^5^ km^2^/My or ∼65% CA/Gy). This rate of lithosphere production is 5 to 20 times the above estimates for global rates of production of transmogrified lithosphere, suggesting that transmogrification, while geologically significant for the growth and replenishment of continents, occurs at only ∼5 to 20% of the long-term rates of crust and lithospheric addition at subduction zones. It would further imply that the rates that Clift et al. (29) inferred for the addition of arc magmas should be reduced by ∼5 to 20% to ∼3 to 3.6 km^3^/y. Note that this relatively high rate of crust/lithospheric production/destruction can still be compatible with the observation that >2.5-Ga cratonic material currently forms a significant fraction of all continents (30) as long as cratonic material is preferentially preserved relative to younger crust/lithosphere additions.

### On the Relative Importance of Transmogrification in Building Continental Crust.

The average Cenozoic river flux exported to the oceans is ∼1.8 km^3^/y (29), close to the ∼1.6-km^3^/y sediment flux estimated to be currently entering global trenches (29). If transmogrified crust diverts ∼38 km × 10^4^ km^3^/My or ∼0.4 km^3^/y of this flux directly back to continental additions, then roughly ∼22% of the riverine sediment flux bypasses subduction to be reaccreted and transmogrified. This makes transmogrification again geologically significant but still a secondary process of continental growth.

### Transmogrification and Seismicity.

**Fig. 4.**
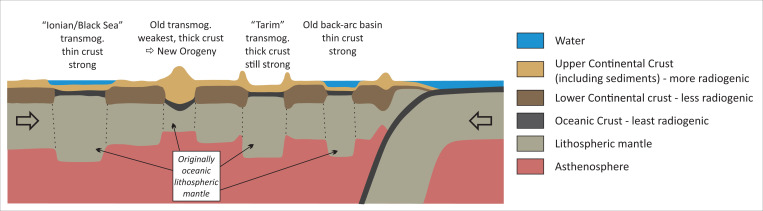
Cartoon showing different “snapshots” of thermal, sedimentary, and mechanical changes that take place during transmogrification; transmog., transmogrified.

During its first ∼500 My of evolution, transmogrified lithosphere will be mechanically stronger than most adjacent lithosphere. Because of this, in regions of continental collision, it would tend to be surrounded by regions of preferred deformation and seismicity. In Asia, this pattern is clearly visible, with magnitude >6 earthquakes surrounding basins with postulated transmogrifying lithosphere (Fig. 1). Note that this inference would imply that seismogenesis is not occurring in transmogrifying lithosphere because it is stronger, not weaker, than surrounding lithosphere. This line of reasoning is also an argument against the strong continental crust/weak continental mantle “crème brulée” hypothesis for the rheology of typical continental lithosphere (31) and in favor of the conventional “jelly sandwich” view that often, but not always (depending on its temperature), the uppermost continental lithospheric mantle is a relatively strong mechanical layer, which is why it is aseismic (32). Our model calculations above assumed that non-Archaean continental and oceanic mantle have the same rheological behavior as a function of pressure, temperature, and strain rate. Transmogrified lithosphere eventually becomes weaker than other types of continental lithosphere because it warms (Figs. 2*F* and 3 *B* and *C*) to the point where it becomes the weakest lithosphere within a continent that has rheologically significant lateral variations in subcontinental crust and mantle temperature (Fig. 4).

### Transmogrification and Epeirogeny.

Because transmogrified crust/lithosphere becomes progressively warmer and weaker and will start to slowly rise ∼100 to 200 My after it reaches >∼30 km in crustal thickness (Figs. 2 *A* and *C* and 3*A*), transmogrification provides an appealing explanation for many of the continental observations of long-lived slow deposition and uplift that led to the early theory of epeirogeny (*cf* [33–35]). In addition to evidence for slow and long-lived ups and downs, such as those seen in Fig. 2*C*, these also include the observed prevalence of deep marine deposits being preferentially exposed in the cores of later mountain belts—for example, that the deep sea marine sediments often exposed in the cores of (tectonically weak) mountain belts were initially deposited on oceanic, not continental, crust.

### Generalized Transmogrification.

The basic concept that a trapped inland basin surrounded by high topography can fill with terrigenous sediments during regional orogenesis to the point where it heats and weakens several 100 My later does not only apply to the ocean crust–floored basins that we focus on here. Any basin that remains cold and strong while being deeply buried by radiogenic sediments will eventually experience burial-induced reheating that causes it to warm, weaken, and thermoisostatically rise. During the Archean, the twofold higher radioactivity of ∼3-Ga rocks would tend to enhance this burial-heating effect, although it has been recently proposed that the Archean crustal heat production was only ∼50% higher due to its more mafic character (36). If plate motions were faster, as many workers have presumed, the average seafloor age (and lithospheric cooling) prior to deep burial would be lower, too, thereby speeding the transition to weak transmogrified crust and lithosphere. This line of speculative reasoning leads us to wonder if Archean transmogrification was the primary mechanism that formed the characteristic Archean crustal planform of greenstone belts—the Archaean oceanic crust—and associated tonalite–trondhjemite–granodiorites (TTGs). In this case, TTGs would be the transmogrified remnants of former Archean sediment infill that partially melted and extensively codeformed with the basalt–protolith greenstones during Archaean orogenies. In contrast, post-Archean, more felsic sediment infill would partially melt to generate granites instead of TTGs. This would rationalize why TTG-like felsic rocks are confined to the Archean with the exception of the remelting of hydrated basalts in unusually hot environments (37), like modern Iceland.

### Transmogrification–Climate Feedbacks.

Transmogrification leads to the formation of relatively strong inland basins during the collisional phase of a Wilson Cycle, such as the present-day Caspian Sea or Tarim Basin, now relatively undeformed and mostly surrounded by orogenic belts. These mountain–belt-bounded inland basins can have a significant impact on regional climate, as the Tarim Basin and its northern bounding Tian Shan mountain belt do today (38). Because the Tarim Basin is mechanically strong, Indo-Asian collision has led to the formation of a pair of high mountain belts to either side of the basin—the Himalaya and the Tian Shan. The particular transmogrification-shaped geomorphology of collisional inland basins surrounded by a pair of high mountain belts is what currently creates a particularly strong East Asian Monsoon that is much stronger than that which would be created by the effect of the Tibetan Plateau alone (38). In turn, the enhanced erosion from the strong monsoon coupled with the inland drainage of Tarim has created a climate and depositional feedback where this inland basin continues to rapidly fill even as it rises. It must continue to fill while it remains deeper than its bounding orogenic belts/Tibetan Plateau until river drainage can break out of the basin, as now happens in the Sichuan Basin with the Yangtze River. This feedback could lead to the generation of particularly thick regions of future transmogrified crust, with associated anomalous deep crustal warming and anatexis. By this end stage of transmogrification, these will behave as relatively weak and ductile regions during rifting or collision.

### Transmogrification and the Formation or Preservation of Large Igneous Provinces.

Finally, if transmogrification leads to eventual warming and weakening at the Moho and underlying lithosphere, it is possible that transmogrified lithosphere could be particularly prone to lithospheric delamination. If aided by the presence of a nearby plume, such delamination could even generate preferred sites for future continental flood basalts and continental rifting (39). Oceanic plateaus, with their thicker/more buoyant than normal crust, may also be preferred candidates for entrapment and transmogrification during continental collision. Note that while it has been suggested that Tarim is underlain by a former ocean plateau (40), or Large Igneous Province (LIP) (41, 42), more recent estimates of oceanic Tarim magmatism (43) seem more consistent with either normal oceanic crust with seamounts or the oceanic seafloor at a volcanic rifted margin. A common thread running through these scenarios is that we should anticipate that the relative strengths of lateral mechanical heterogeneities in the continental lithosphere could change through time.

### Seismic Tests.

Seismic tools currently exist to better test the proposed transmogrification of ocean basins into continent, and this hypothesis highlights our continued need to explore the deep seismic structure of continental crust. For example, a well-constrained seismic search for the presence of former ocean crust above the Moho of the Tarim Basin still needs to be done. We hope that this work will motivate future researchers to perform this and other tests that will further stimulate new thoughts on the long-term evolution of continents and on potential interactions between plate tectonics and climate.

## Materials and Methods

### Numerical Methods.

The numerical experiments in this study use a numerical code written in MATLAB (https://www.mathworks.com) that solves a 1D transient heat conduction equation to capture the evolving thermal structure of ocean crust that is progressively buried by continental sediments. Transient heat conduction is modeled using a 1D control volume approximation with finite difference approximations used to determine the heat fluxes entering and leaving each control volume. A standard Crank–Nicolson approximation is used for the time derivative. After each timestep, sediment is added to the top of the region by moving the top grid point upwards. A new mesh point is added (with a linearly interpolated initial temperature) whenever the spacing between the top node and its underlying node exceeds 2 km. Below the topmost two grid points, grid points are uniformly spaced every 1 km.

The initial thermal structure is taken to be that of an idealized midocean ridge axis with 7-km–thick oceanic crust and mantle, all initially at the mantle temperature except for the top node, which is kept at 0 °C—also used to start a similar half-space analytical cooling solution that the code was benchmarked to <1 °C accuracy for a 1-km grid spacing. The bottommost grid point at a 250-km depth is fixed at 1,250 °C. Radioactive heat production in continental sediments, ocean crust, and mantle is assumed to be 9.63 × 10^−10^ W/kg, 1.77 × 10^−11^ W/kg, and 7.38 × 10^−12^ W/kg, respectively (44).

Isostatic relief was determined assuming that only the sediments compact and densify with increasing pressure, while sediments, oceanic crust, and mantle all have a temperature-dependent density. The sedimentary density versus pressure dependence is assumed to follow that of Curray’s measured density depth distribution in the Bengal Fan (45, 46). Isostatic relief is determined by integrating the density of each column and comparing its predicted isostatic depth to that of an initial sediment-free midocean ridge column that is assumed to correspond to a depth of 2,700 m (for a similar implementation see appendix C of ref. 47). Surface heat flow is calculated using the temperature gradient at the top of the column and a thermal conductivity of 3 W m^−1^ °C^−1^ (44). Rheological profiles use the temperature, rock composition (sediment, ocean crust, or mantle), and an assumed horizontal strain rate of 10^−15^ s^−1^ to determine the yield stress envelope following the methodology of Kohlstedt et al. (21) and using Mohr–Coulomb and Goetze’s criteria brittle failure and the creep parameterizations in the implementation of Ros et al. (20). The creep parameters used here are “wet olivine” (48) for lithospheric and asthenospheric mantle, “mafic granulite” (49) for oceanic crust, and “wet quartzite” (50) for sediments/transmogrifying crust. These parameterizations and parameter values are found in the MATLAB subfunction Calc_YSE. All MATLAB codes used in the above calculations are included as Codes S1–S5.

Fig. 3D combines results from two numerical experiments. The Ionian Sea–like and Tarim Basin–like evolution of transmogrification involves 10 km of initial basin fill over the first 250 My of evolution (mimicking the Ionian Sea history shown in figure 12 of ref. 7), followed by 24 km of sedimentation over the next 150 My. The Black Sea–like and Caspian Sea–like evolution of transmogrification involves 7 km of initial basin fill over the first 110 My followed by 13 km of sedimentation over the next 34 My, following the Caspian Sea sedimentation history as shown in the stratigraphic column in figure 2 of ref. 10. All other parameters are as in the above exemplar calculation.

## Supplementary Material

Supplementary File

Supplementary File

Supplementary File

Supplementary File

Supplementary File

## Data Availability

All study data are included in the article and/or supporting information.
